# The Influence of Different Implant Placement Techniques on Alveolar Ridge Preservation: A Systematic Review and Meta-analysis

**DOI:** 10.1055/s-0045-1806862

**Published:** 2025-04-23

**Authors:** Nguyen Phu Thang, Nguyen Thi Khanh Ly, Do Thi Thanh Toan, Nguyen Thu Tra, Nguyen Minh Duc

**Affiliations:** 1 Department of Oral Surgery, School of Dentistry, Hanoi Medical University, Hanoi, Vietnam; 2 Department of Medical Statistics and Informatics, Institute for Preventive Medicine and Public Health, Hanoi Medical University, Hanoi, Vietnam; 3 Department of Prosthodontics, School of Dentistry, Hanoi Medical University, Hanoi, Vietnam; 4 Division of Research and Treatment for Oral Maxillofacial Congenital Anomalies, Aichi Gakuin University, Nagoya, Japan

**Keywords:** alveolar bone, socket shield, immediate implant placement

## Abstract

This systematic review and meta-analysis compares the effectiveness of three implant placement techniques: socket shield technique (SST), conventional immediate implant placement (CIIP), and delayed implant placement (DIP) in alveolar ridge preservation, implant survival rates, and esthetics. A comprehensive search was conducted in PubMed, Scopus, and the Cochrane Library, covering studies from 2012 to 2022. Inclusion criteria targeted clinical studies with a minimum follow-up of 6 months. Risk of bias was assessed using RoB-2 and ROBINS-I tools, and meta-analyses were performed using random-effects models. Sixteen studies met the inclusion criteria. SST demonstrated significantly better preservation of buccal bone thickness (standardized mean difference [SMD] = 2.94, 95% confidence interval [CI]: 1.46–4.42, *p* < 0.001) and height (SMD = 4.47, 95% CI: 1.96–6.98, *p* < 0.001) compared with CIIP. SST also resulted in higher pink esthetic scores (SMD = 1.00, 95% CI: 0.36–1.64, *p* = 0.002). No significant differences were found between CIIP and DIP for marginal bone loss (SMD = 0.15, 95% CI: −0.26 to 0.55, *p* = 0.471). However, DIP showed a lower implant failure rate than CIIP (odds ratio = 3.49, 95% CI: 1.26–9.66, *p* = 0.016). SST offers significant benefits in preserving alveolar bone and improving esthetic outcomes, while DIP appears to reduce implant failure risk. Further standardized studies are needed to confirm these findings and refine clinical guidelines.

## Introduction

Tooth extraction frequently leads to alveolar ridge resorption, which can compromise future implant placement and esthetic outcomes. This resorptive process affects both vertical and horizontal bone dimensions, posing a challenge for successful implant treatment. 1 Selecting an appropriate implant placement technique is critical for maintaining bone integrity and achieving long-term functional and esthetic success.

Over the years, various implant placement approaches have been developed to mitigate the adverse effects of bone loss while promoting favorable outcomes for implant placement. socket shield technique (SST), which preserves part of the tooth root to maintain buccal bone and soft tissue architecture, has gained attention for its potential to enhance both esthetic outcomes and implant survival rates. 2 3 Delayed implant placement (DIP), by contrast, allows for a healing period after extraction before implant placement, facilitating improved osseointegration and greater long-term stability, particularly in cases of significant bone loss or infection. 4 Finally, conventional immediate implant placement (CIIP), which involves placing the implant immediately after extraction, provides potential benefits such as reduced treatment timelines and preserved bone volume, though its long-term efficacy in preserving alveolar ridge dimensions is still under investigation. 5

Nevertheless, much of the existing literature consists of case reports and small clinical trials, which often lack robust clinical controls and clinically relevant outcomes. This inconsistency in study designs and results is also reflected in previous systematic reviews and meta-analyses, posing a significant challenge in synthesizing reliable evidence. For example, Pickert et al conducted a meta-analysis evaluating bone graft materials and found that nonautologous grafts had a greater effect on reducing bone resorption compared with autologous materials. However, their study did not evaluate the effectiveness of implant placement techniques in alveolar ridge preservation (ARP), which limits the applicability of their findings. 6

This systematic review and meta-analysis aims to evaluate the comparative effectiveness of SST, DIP, and CIIP in terms of post-extraction dimensional changes, implant survival rates, and pink esthetic scores (PESs). Through a comprehensive assessment, this review seeks to provide an evidence-based understanding of the strengths and limitations of each technique, thereby offering guidance for clinicians to make informed decisions when selecting the most appropriate implant placement approach.

## Methods

### Objective

This systematic review adhered to the Preferred Reporting Items for Systematic Reviews and Meta-Analyses (PRISMA) statement and guidelines provided by the Cochrane Handbook for Systematic Reviews to minimize the number of missing articles and enhance the clarity and transparency of the systematic review. 7 The focused research question was developed using the PICO format (Population, Intervention, Control, Outcome), 8 as follows: What are the marginal bone loss, changes in buccal bone width (BBW), PES, and dental implant failure rate from three implant placement techniques: SST, CIIP, and DIP? (P) Population: patients who underwent implant placement following tooth extraction with a minimum follow-up of 6 months; (I) Intervention: SST or CIIP or DIP; Comparison (C): comparison among SST, CIIP, and DIP; (O) Outcomes: primary outcomes included changes in buccal bone thickness and height, and marginal bone loss. Secondary outcomes were PES and implant failure rate.

### Search Strategy

An electronic search was conducted across PubMed (MEDLINE), Scopus, and Cochrane Library databases using specific search strings. The search was limited to articles published between January 1, 2012 and December 31, 2022. The final search update was performed in January 2023. Studies retrieved from the search were imported into EndNote X9 (Clarivate Analytics, Pennsylvania, United States) for organization and management. The search terms used for each database are listed in **Appendix A1** .

### Inclusion and Exclusion Criteria

Eligibility criteria included randomized controlled trials (RCTs) and nonrandomized prospective clinical trials (PCTs) with a minimum follow-up period of 6 months, full-text articles published between 2012 and 2022, and reporting qualitative and quantitative parameters of buccal bone thickness and height, marginal bone loss, PES, and implant failure rate. Included studies assessed implant placement in both esthetic and nonesthetic zones, depending on the study focus. Studies not published in English, systematic reviews, case reports, case-control, technical reports, finite element analyses, animal studies, and review articles were excluded.

### Quality Assessment

The included studies were independently screened by two examiners (N.P.T. and N.T.K.L.). Titles and abstracts of all studies retrieved in the online search were first scanned, and the full text of studies that met the inclusion criteria was obtained. Full-text versions of studies that did not provide sufficient data in the title and abstract were also reviewed to make a final decision regarding eligibility. Any differences were addressed collaboratively to achieve agreement among all authors. The RoB-2 tool was used to assess the risk of bias in RCTs, 9 and the ROBINS-I tool was used to evaluate the risk of bias in non-RCTs. 10 The risk of bias was categorized as “high,” “low,” or “unclear” for each item, and the overall risk was classified as high, moderate, or low.

### Quantitative Synthesis—Meta-analysis

The studies included in the meta-analysis were combined using a random-effects model, with various methods used to estimate effect size. The inverse variance method was applied to estimate continuous outcomes, while the Mantel–Haenszel method was used for dichotomous outcomes. All variables were expressed with 95% confidence intervals (CIs). Heterogeneity between studies was assessed using the Q-test ( *p* -value < 0.05) and quantified with the *I* ^2^ statistic, with slight heterogeneity considered between 25 and 50%, moderate between 50 and 75%, and high above 75%. Statistical significance was assessed using the Z-test ( *p* -value < 0.05). Meta-analysis results were presented in forest plots, and publication bias was assessed using the trim-and-fill method, with results displayed in funnel plots. 11

Statistical analyses were conducted using Review Manager Software (RevMan Version 5.3, the Nordic Cochrane Centre, Cochrane Collaboration, Copenhagen, Denmark), and STATA 14 software (Stata Corporation, College Station, Texas, United States) for additional statistical assessments.

## Result

### Flow Diagram

**Fig. 1:**
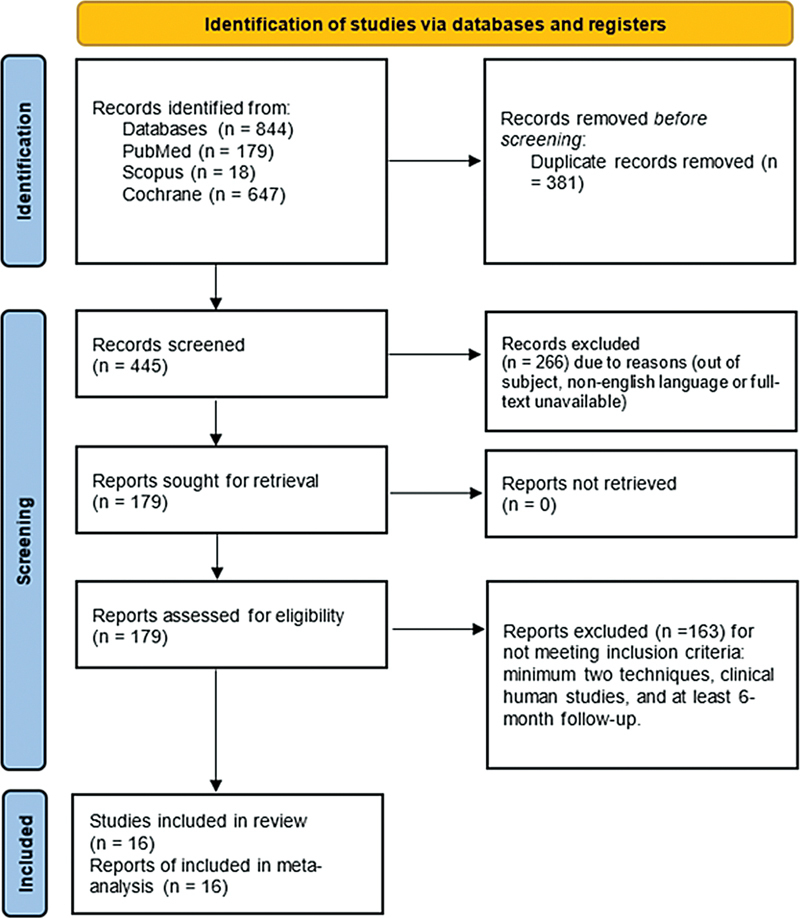
Flow diagram depicting the selection of studies in accordance with PRISMA guidelines.

The initial electronic search was performed in January of 2023: 179 articles in PubMed and 647 in Cochrane, and 18 articles from Scopus. Of the total of 844 studies, 381 were discarded due to being duplicates and 266 were discarded because of lack of abstract or full texts or using other languages rather than English. After screening the titles and abstracts, a further 163 were rejected, as they failed to fulfill the following inclusion criteria: using at least two techniques, clinical human studies, and presenting a minimum follow-up time of 6 months. A final total of 16 articles were included in the qualitative and quantitative synthesis, as these included all the data and variables required ( Fig. 1 ).

### Qualitative Analysis

Of the 16 articles included, 11 were RCTs, 12 13 14 15 16 17 18 19 20 21 22 and 5 were PCTs. 23 24 25 26 27 The sample sizes of the studies selected in the present meta-analysis range from 10 in the study by Mathew et al 26 to 196 in Esposito et al's study, 18 with the subject ages ranging from 18 years old 15 to 72 years old, 20 and the follow-up times from 6 months 12 23 to 36 months. 13 19 22 The results are presented in Table 1 .

**Table 1:** General characteristics of included studies

No.	Authors, year	Country	Type of study	Follow-up period	Sample ( *n* )	Dental implant failure rate	Margin bone loss	Pink esthetic score	Horizontal bone loss	Vertical bone loss
1	Abd-Elrahman et al, 2020 12	Egypt	RCT	6 months	40 dental implants (25 patients 21–39 years old)	0/20 CIIP 0/20 SST		CIIP: 8.85 ± 1.81 SST: 12.0 ± 1.12	CIIP: 0.28 ± 0.15 mm SST: 0.12 ± 0.07 mm	CIIP: 0.77 ± 0.35 mm SST: 0.34 ± 0.12 mm
2	Barakat et al, 2017 23	Egypt	PCT	6 months	20 dental implants (20 patients 20–50 years old)	0/10 CIIP 0/10 SST			CIIP: 0.34 ± 0.11 mm SST: 0.1 ± 0.03 mm	CIIP: 1.61 ± 0.78 mm SST: 0.44 ± 0.24 mm
3	Bramanti et al, 2018 13	Italy	RCT	36 months	40 dental implants (40 patients)	0/20 CIIP 0/20 SST	CIIP: 1.11 ± 0.13 mm SST: 0.60 ± 0.06 mm	CIIP: 10.30 ± 2.53 SST: 12.15 ± 0.76		
4	Fattouh, 2018 24	Egypt	PCT	12 months	20 dental implants (20 patients)	0/10 CIIP 0/10 SST		CIIP: 10.3 ± 0.48 SST: 11.2 ± 0.91		
5	Hana & Omar, 2020 25	Iraq	PCT	12 months	40 dental implants (40 patients 28–65 years old)	1/20 CIIP 1/20 SST				
6	Sun et al, 2020 14	China	RCT	24 months	30 dental implants (30 patients)	0/15 CIIP 0/15 SST	NAv	CIIP: 11.73 ± 1.76 SST: 12.07 ± 1.62	CIIP: 0.53 ± 0.05 mm SST: 0.22 ± 0.09 mm	CIIP: 0.87 ± 0.12 mm SST: 0.28 ± 0.08 mm
7	Tiwari et al, 2020 15	India	RCT	12 months	16 dental implants (16 patients)	0/8 CIIP 0/8 SST	CIIP: 0.188 ± 0.013 mm SST: 0.030 ± 0.025 mm		CIIP: 0.19 ± 0.09 mm SST: 0.03 ± 0.13 mm	CIIP: 0.16 ± 0.01 mm SST: 0.03 ± 0.01 mm
8	Mathew et al, 2020 26	United States	PCT	12 months	10 dental implants (10 patients 25–60 years old)	N/A		CIIP: 10.8 ± 0.84 SST: 12.2 ± 0.837		
9	Santhanakrishnan et al, 2021 16	India	Prospective RCT	12 months	75 dental implants (75 patients 18–50 years old)	0/25 CIIP 0/25 SST 0/25 DIP		CIIP: 11.2 ± 2.10 SST: 11.7 ± 1.8 DIP: 10.2 ± 1.4	CIIP: 0.4 ± 0.1 mm SST: 0.05 ± 0.02 mm DIP: 0.2 ± 0.1 mm	
10	Tallarico et al, 2017 17	Italy	RCT	12 months	24 dental implants (24 patients 37–67 years old)	0/12 CIIP 0/12 DIP	CIIP: 0.23 ± 0.2 mm DIP: 0.12 ± 0.23 mm	CIIP: 10.6 ± 1.8 DIP: 12.2 ± 1.2		
11	Esposito et al, 2017 18	Italy	RCT	12 months	131 dental implants (131 patients 20–50 years old)	4/67 CIIP 1/64 DIP	CIIP: 0.25 ± 0.17 mm DIP: 0.31 ± 0.16 mm	CIIP: 12.52 ± 1.08 DIP: 11.78 ± 1.1		
12	Checchi et al, 2017 19	France	RCT	12 months	91 dental implants (91 patients)	5/47 CIIP 2/44 DIP		CIIP: 9.71 ± 2.71 DIP: 10.86 ± 1.37		
13	Esposito et al, 2015 20	Italy	RCT	12 months	106 dental implants (106 patients 28–72 years old)	2/54 CIIP 0/52 DIP	CIIP: 0.29 ± 0.15 mm DIP: 0.23 ± 0.11	CIIP: 13.0 ± 1.5 DIP: 12.8 ± 1.4		
14	Felice et al, 2015 21	Italy	RCT	12 months	50 dental implants (50 patients 32–70 years old)	2/25 CIIP 0/25 DIP	CIIP: 0.25 ± 0.15 mm DIP: 0.15 ± 0.1 mm	CIIP: 12.78 ± 1.09 DIP:12.22 ± 1.13		
15	Raes et al, 2013 27	Belgium	PCT	12 months	39 dental implants (39 patients 20–69 years old)	1/16 CIIP 0/23 DIP	CIIP: 0.46 ± 1.84 mm DIP: 0.53 ± 0.89 mm	CIIP: 10.33 ± 2.29 DIP:10.11 ± 1.9		
16	Cucchi et al, 2017 22	Italy	RCT	36 months	97 dental implants (92 patients 18–30 years old)	2/49 CIIP 0/48 DIP	CIIP: 0.4 ± 0.4 mm DIP: 0.4 ± 0.4 mm			

Abbreviations: CIIP, conventional immediate implant placement; DIP, delayed implant placement; PCT, prospective clinical trial; RCT, randomized controlled trial; SST, socket shield technique.

### Quality Assessment

The RoB-2 tool was used to assess the risk of bias in the human RCT, and the results are presented in Table 2 . Nine studies had a low risk of bias, and two studies had a moderate risk of bias. The ROBINS-I tool was used to evaluate the risk of bias in the non-RCTs. A detailed evaluation of the possible risk of bias for all categories is summarized in Table 2 .

**Table 2:** The risk of bias of included studies

**ROBINS-I**							
**Nonrandomized trials**	**Confounding**	**Subject selection**	**Classification of interventions**	**Protocol deviations**	**Missing data**	**Outcome measurement**	**Reporting**
Barakat et al, 2017 23	Low	Low	Low	Moderate	Low	Low	Low
Hana & Omar, 2020 25	Unclear	Low	Low	Low	Low	Low	Low
Mathew et al, 2020 26	Unclear	Low	Low	Low	Low	Low	Low
Raes et al, 2013 27	Low	Low	Low	Low	Low	Low	Low
Fattouh, 2018 24	Low	Low	Low	Low	Low	Low	Low
**RoB-2**							
**Randomized trials**	**Confounding**	**Subject selection**	**Classification of interventions**	**Protocol deviations**	**Missing data**	**Outcome measurement**	**Reporting**
Abd-Elrahman et al, 2020 12	Low	Low	Low	Low	Low		
Bramanti et al, 2018 13	Low	Low	Low	Low	Low		
Sun et al, 2020 14	Low	Low	Low	Low	Low		
Tiwari et al, 2020 15	Unclear	Low	Low	Low	Moderate		
Santhanakrishnan et al, 2021 16	Low	Low	Low	Low	Low		
Tallarico et al, 2017 17	Unclear	Low	Low	Moderate	Low		
Esposito et al, 2017 18	Unclear	Low	Low	Low	Low		
Felice et al, 2015 21	Low	Low	Moderate	Moderate	Moderate		
Cucchi et al, 2017 22	Low	Low	Low	Low	Low		
Checchi et al, 2017 19	Low	Low	Low	Low	Low		
Esposito et al, 2015 20	Low	Low	Low	Low	Low		

### Quantitative Analysis

#### Bone Changes

##### Buccal Bone Thickness

###### SST versus CIIP

Five studies assessed changes in the thickness of the buccal bone plate using the SST and CIIP methods, involving a total of 156 patients (78 SST patients and 78 CIIP patients). 12 14 15 16 23 A meta-analysis using the random-effects model showed that the SST method resulted in less buccal bone resorption compared with the CIIP method. The difference was statistically significant, with a standardized mean difference (SMD) of 2.94 (95% CI: 1.46–4.42, *p* < 0.001). The average change in buccal bone thickness was −0.105 mm for the SST method and −0.365 mm for the CIIP method.

**Fig. 2:**
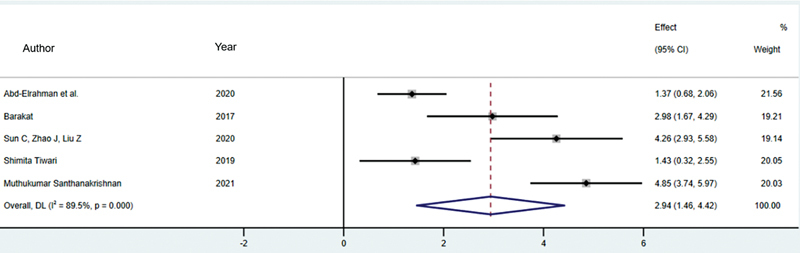
Comparison of buccal bone thickness changes between SST versus CIIP. CIIP, conventional immediate implant placement; SST, socket shield technique.

The analysis revealed high heterogeneity, with an *I* ^2^ of 89.5% ( *p* < 0.001; Fig. 2 ).

##### Buccal Bone Height

###### SST versus CIIP

**Fig. 3:**
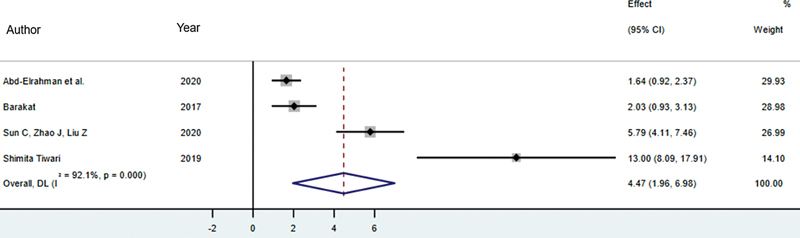
Comparison of buccal bone heigh changes between SST versus CIIP. CIIP, conventional immediate implant placement; SST, socket shield technique.

Four studies assessed changes in buccal bone height using the SST and CIIP methods, involving a total of 106 patients (53 SST patients and 53 CIIP patients). 12 14 15 23 A meta-analysis showed that the SST method resulted in less reduction in buccal bone height compared with the CIIP method. The difference was statistically significant, with an SMD of 4.47 (95% CI: 1.96–6.98, *p* < 0.001). The average change in buccal bone height was −0.30 mm for the SST method and −0.86 mm for the CIIP method. The *I* ^2^ of 92.1% ( *p* < 0.001) indicates significant heterogeneity among the studies ( Fig. 3 ).

##### Marginal Bone Loss

###### SST and CIIP

**Fig. 4:**
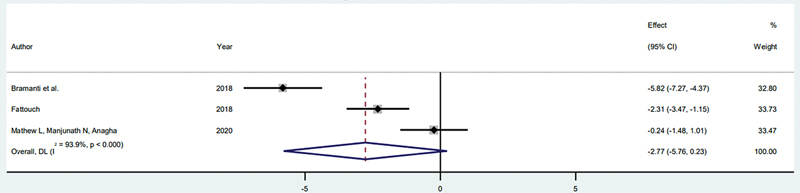
Comparison of marginal bone loss between SST and CIIP. CIIP, conventional immediate implant placement; SST, socket shield technique.

Three studies assessed marginal bone loss using the SST and CIIP methods, involving a total of 70 patients (35 SST patients and 35 CIIP patients). 13 24 26 A meta-analysis using the random-effects model indicated that peri-implant bone resorption tended to be lower in the SST group compared with the CIIP group. However, the difference was not statistically significant, with an SMD of −2.769 (95% CI: −5.76 to 0.23, *p* = 0.07). The analysis also revealed high heterogeneity, with an *I* ^2^ of 93.9% ( *p* < 0.001; Fig. 4 ).

###### CIIP and DIP

**Fig. 5:**
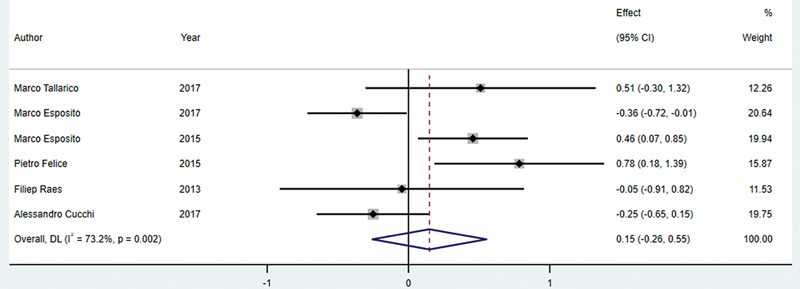
Comparison of marginal bone loss between CIIP and DIP. CIIP, conventional immediate implant placement; DIP, delayed implant placement.

The forest plot compares marginal bone loss between CIIP and DIP across six studies with a total of 223 patients in the CIIP group and 220 in the DIP group. 17 18 20 21 22 27 The estimated mean difference is 0.15 mm (95% CI: −0.26 to 0.55), with a *p* -value of 0.471, indicating no statistically significant difference between the two methods. The heterogeneity among the studies is moderate, with *I* ^2^ = 73.2% ( *p* = 0.002; Fig. 5 ).

#### Pink Esthetic Score

##### CIIP and SST

**Fig. 6:**
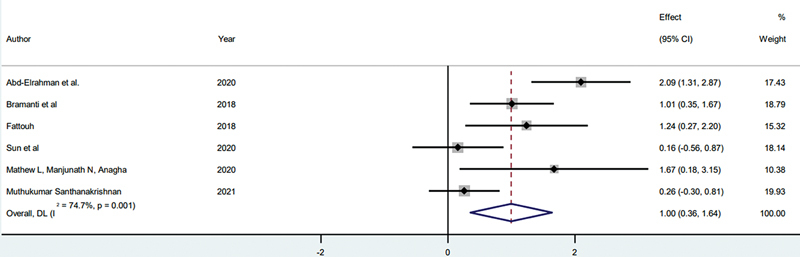
Comparison of PES between CIIP and SST. CIIP, conventional immediate implant placement; PES, pink esthetic score; SST, socket shield technique.

The meta-analysis of PESs from six studies 12 13 14 16 24 26 comparing CIIP ( *n* = 95) and SST ( *n* = 95), with follow-up periods ranging from 12 to 36 months, shows a SMD of 1.00 (95% CI: 0.36–1.64, *p* = 0.002), favoring SST for improved esthetic outcomes. Study weights vary, with Abd-Elrahman et al 12 and Bramanti et al 13 contributing the most. Moderate heterogeneity ( *I* ^2^ = 74.7%, *p* = 0.001) indicates some inter-study variability, which may affect the pooled effect size ( Fig. 6 ). The average PES for the SST method is 11.9, and for the CIIP method, it is 10.6

##### CIIP and DIP

**Fig. 7:**
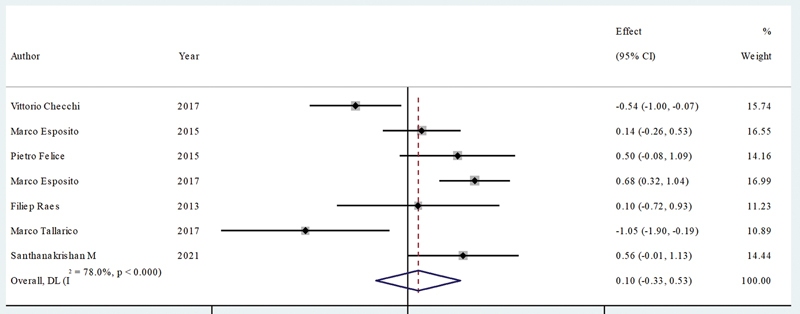
Comparison of PES for the CIIP and DIP. CIIP, conventional immediate implant placement; DIP, delayed implant placement; PES, pink esthetic score.

Seven studies compared the PESs for the CIIP ( *N* = 246) and DIP ( *N* = 233) with total 479 implants. 16 17 18 19 20 21 27 The estimated mean difference was 0.1 mm (95% CI: −0.33 to 0.53) and statistically insignificant ( *p* = 0.591), and the heterogeneity was medium according to the meta-analysis ( *I* ^2^ = 78%, *p* < 0.001; Fig. 7 ).

#### Failure Rate

##### CIIP versus DIP

**Fig. 8:**
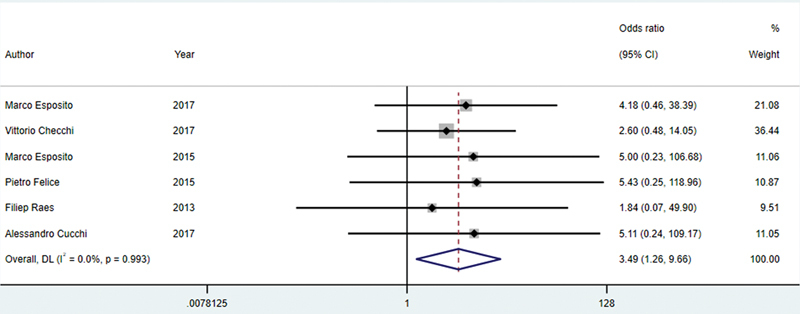
Comparison of failure rate for the CIIP and DIP. CIIP, conventional immediate implant placement; DIP, delayed implant placement.

A meta-analysis of implant failure rates from six studies 18 19 20 21 22 27 with 514 patients comparing CIIP ( *N* = 258) and DIP ( *N* = 256) yielded a pooled odds ratio of 3.49 (95% CI: 1.26–9.66), *p* = 0.016, indicating a statistically significant increase in failure risk for CIIP compared with DIP ( Fig. 8 ). The heterogeneity among studies was low ( *I* ^2^ = 0.0%, *p* = 0.993), indicating consistent findings across studies.

## Discussion

This systematic review and meta-analysis evaluated the effectiveness of three implant placement techniques, SST, CIIP and DIP, in maintaining bone dimensions, enhancing soft tissue esthetics, and improving implant success. An appropriate implant placement technique is crucial for ARP, but there is no clear consensus on the most effective approach. Seventeen studies, including RCTs and PCTs with a minimum follow-up of 6 months, met the inclusion criteria, ensuring methodological consistency and allowing for a robust comparison of implant placement techniques.

Previous reviews, such as those by Avila-Ortiz et al 28 and Aribau-Gumà et al 29 also examined ARP methods but differed in study design and selection criteria. Avila-Ortiz et al focused only on RCTs and highlighted the need for long-term data, while Aribau-Gumà et al included studies of varying quality, noting inconsistencies across findings. This review expands on prior research by combining both randomized and prospective trials, offering a broader perspective on ARP effectiveness, though variability in study designs remains a limitation.

### Changes in Buccal Bone Thickness and Height

#### SST versus CIIP

This meta-analysis highlights that the SST significantly preserves both buccal bone thickness and height compared with CIIP, with SMD of 2.94 and 4.47, respectively. SST's average reductions in thickness (−0.105 mm) and height (−0.30 mm) were substantially lower than those observed with CIIP (−0.365 mm for thickness and −0.86 mm for height).

The advantage of the SST in preserving buccal bone thickness and height is likely attributed to its approach of partially retaining the tooth root, which helps maintain the structural integrity of the surrounding bone. By preserving a portion of the root, SST reduces the likelihood of bundle bone loss—a common consequence of tooth extraction—since bundle bone relies on the presence of a tooth root for its vascular and structural support. 30 This preservation of the buccal plate mitigates the resorptive processes that typically occur post-extraction, thereby maintaining both the horizontal and vertical dimensions of the alveolar ridge.

However, the technique is not without its challenges. SST demands a high degree of surgical precision and skill, as the retained root fragment must be handled carefully to prevent issues such as mobility or dislodgement, which could destabilize the implant or compromise healing. Furthermore, leaving a portion of the root in place carries a risk of infection if remnants of pulp tissue or bacteria remain within the fragment, potentially leading to complications like cyst formation or peri-implantitis. 31 These complexities make SST a technically demanding procedure, requiring practitioners with advanced skills and experience to perform it effectively. Consequently, SST is best suited for highly skilled clinicians and in cases where the benefits of preserving alveolar bone and soft tissue contours—such as in the esthetic zone—outweigh the procedural risks.

The high heterogeneity in the studies ( *I* ^2^ = 89.5% for thickness and 92.1% for height), likely due to differences in protocols, patient characteristics, and measurement techniques, underscores the need for more standardized research. Additionally, potential publication bias may influence the results, as studies with positive outcomes for SST are often more likely to be published. While SST shows promise for preserving bone and esthetics, particularly in the esthetic zone, further large-scale, long-term studies are required to confirm its clinical efficacy and define its role in practice.

### Marginal Bone Loss

#### SST versus CIIP

The analysis shows that SST tends to reduce marginal bone loss more effectively than CIIP, with an SMD of −2.77, although this difference was not statistically significant ( *p* = 0.07). SST's potential to preserve marginal bone aligns with its effects on buccal bone height and thickness, where it has been shown to maintain bone thickness and height better than CIIP. This broader preservation of both marginal bone and buccal dimensions highlights SST's ability to support structural stability and esthetic outcomes. However, the high heterogeneity ( *I* ^2^ = 93.9%) in these findings suggests variability in study designs and patient characteristics, emphasizing the need for more standardized research to confirm SST's overall advantages in both marginal and buccal bone preservation.

#### CIIP versus DIP

The comparison between CIIP and DIP reveals minimal difference in marginal bone loss, with a mean difference of 0.15 mm. This suggests that, in terms of bone preservation, the two methods perform similarly and may be selected based on other clinical considerations rather than bone loss outcomes. In CIIP, the immediate placement of the implant takes advantage of the fresh extraction socket, potentially reducing initial bone loss. However, some bone remodeling, particularly in the buccal plate, is still expected. Conversely, DIP allows for socket healing before placement, potentially providing more stable integration but also permitting minor bone resorption during the delay. 32 The moderate heterogeneity ( *I* ^2^ = 73.2%) among studies likely reflects variations in patient characteristics, surgical techniques, and follow-up periods, indicating that factors beyond timing alone, such as bone quality and implant type, may influence outcomes. These findings suggest that both CIIP and DIP offer comparable results for marginal bone preservation, with the choice between them best guided by individual clinical considerations. Further standardized research is needed to refine guidelines for their optimal use.

### Pink Esthetic Scores

#### CIIP versus SST

The PES analysis indicates a significant esthetic advantage for SST over CIIP, with SST achieving an average PES of 11.9 compared with 10.6 for CIIP ( *p* = 0.002). This aesthetic advantage is probably due to SST's capability to maintain the buccal bone by partially retaining the tooth root, which offers structural support to both the buccal bone and the adjacent soft tissues. By maintaining this structure, SST reduces the likelihood of gingival collapse and recession, essential for preserving the natural contour in esthetically critical areas, such as the anterior maxilla. Small changes in soft tissue volume can be particularly noticeable in these regions, making SST a valuable technique for maintaining an appealing appearance post-implant.

The moderate heterogeneity ( *I* ^2^ = 74.7%) among studies suggests some variability in results, likely due to differences in study designs, follow-up durations, and PES assessment methods. Despite this variability, the overall trend strongly favors SST, indicating it may be the preferred option for cases where esthetics are a priority. However, the presence of moderate heterogeneity implies that while the findings are promising, further standardized studies would help to validate SST's esthetic advantages more consistently across diverse clinical settings.

#### CIIP versus DIP

The comparable esthetic outcomes between CIIP and DIP, with a small SMD of 0.1 and an insignificant *p* -value, suggest that neither approach provides a distinct advantage for maintaining soft tissue contours. This allows clinicians to base their choice on other factors, such as timing preferences or specific clinical conditions. The similar esthetic outcomes between CIIP and DIP may stem from natural bone remodeling that occurs post-extraction, particularly in the buccal plate. In both methods, some resorption is inevitable due to the loss of bundle bone support after tooth extraction, which impacts soft tissue contours over time. This suggests that factors beyond timing, like patient-specific bone quality and buccal bone thickness, might play a more significant role in determining esthetic results, underscoring the importance of individualized treatment planning. Standardizing protocols in future research would help reduce this variability and provide clearer guidance on when each technique might be preferable in esthetic-sensitive cases.

### Implant Failure Rate

#### CIIP versus DIP

The meta-analysis indicates a significant increase in implant failure risk with CIIP compared with DIP, as reflected by a pooled odds ratio of 3.49 (95% CI: 1.26–9.66, *p* = 0.016). Challenges with osseointegration in fresh extraction sockets can compromise the stability required for successful implant integration with CIIP. While immediate placement aims to reduce bone loss, it may not adequately address the compromised quality of surrounding bone, hindering long-term stability. 33

The low heterogeneity ( *I* ^2^ = 0.0%) among the studies supports the reliability of these results, indicating consistent findings across different populations and clinical settings. This analysis emphasizes the importance of considering implant placement timing in clinical decision-making. CIIP may offer advantages in terms of reduced treatment time; the increased risk of failure suggests that practitioners should carefully evaluate individual patient factors and the specific clinical context before choosing the optimal approach. Further research is warranted to clarify the mechanisms contributing to the higher failure rates associated with CIIP, ultimately informing better protocols in implant dentistry.

### Limitations

This study has several limitations that should be considered when interpreting the findings. First, we relied on data reported in the included studies, meaning we had no control over the measurement methodologies used for outcomes such as BBW, height, and marginal bone loss. Variations in measurement techniques, reference points, and reporting standards across studies may have introduced inconsistencies in the pooled data. Second, some analyses included a limited number of studies, reducing statistical power and generalizability. This also impacted the reliability of publication bias assessment, as certain methods require a sufficient number of studies for accurate detection.

Future research should focus on conducting large-scale, standardized RCTs to reduce heterogeneity and improve the comparability of SST, CIIP, and DIP. Long-term follow-up studies (≥5 years) are needed to assess implant survival, bone stability, and esthetic outcomes over time. Additionally, consistent measurement protocols for ARP should be established to enhance data reliability. Further meta-analyses should incorporate a larger number of studies to enable more robust publication bias assessment.

## Conclusion

This review and meta-analysis shows that SST offers significant benefits in preserving buccal bone and improving esthetic outcomes compared with CIIP. While CIIP remains common, DIP demonstrates a lower implant failure rate, suggesting it as a reliable option for long-term stability. Each implant placement technique has unique strengths and limitations, highlighting the importance of tailoring the approach to individual patient needs and clinical goals. Further standardized research is needed to confirm these findings and enhance guidelines in implant dentistry.

## Notes

Appendix A1
**PubMed**
((“socket shield”[Title/Abstract] OR “immediate implant placement”[Title/Abstract] OR “delayed implant placement”[Title/Abstract] OR “alveolar ridge preservation”[Title/Abstract] OR “ridge preservation”[Title/Abstract]) AND (“tooth extraction”[Title/Abstract] OR “tooth removal”[Title/Abstract] OR “extraction”[Title/Abstract] OR “dental extraction”[Title/Abstract] OR “extraction tooth”[Title/Abstract])) AND ((clinicaltrial[Filter] OR randomizedcontrolledtrial[Filter]) AND (2012:2022[pdat]))
**Cochrane**
#1 (tooth extraction):ti,ab,kw OR (tooth removal):ti,ab,kw OR (extraction):ti,ab,kw OR (dental extraction):ti,ab,kw OR (extraction, tooth):ti,ab,kw #2 (socket shield):ti,ab,kw OR (immediate implant placement):ti,ab,kw OR (delayed implant placement):ti,ab,kw OR (alveolar ridge preservation):ti,ab,kw OR (ridge preservation):ti,ab,kw #3 #1 and #2
**Scopus**
(TITLE-ABS-KEY (socket AND shield OR immediate AND implant AND placement OR delayed AND implant AND placement OR alveolar AND ridge AND preservation OR ridge AND preservation) AND TITLE-ABS-KEY (tooth AND extraction OR tooth AND removal OR extraction OR dental AND extraction OR extraction AND tooth) AND TITLE-ABS-KEY (clinical AND trial OR randomized AND controlled AND trial)) AND (LIMIT-TO (SUBJAREA, “DENT”)) AND (LIMIT-TO (LANGUAGE, “English”)).
